# Integrated analysis of phenotypic and SSR data reveals the genetic structure differentiation of wild *Rhododendron mariae* Hance populations in Guangdong and the driving factors for conservation planning

**DOI:** 10.3389/fpls.2026.1723036

**Published:** 2026-03-23

**Authors:** Xin Yan, Jixia Guo, Yongyin Zhang, Xiner Ye, Shengjie Yang, Quan Yang, Xilong Zheng

**Affiliations:** 1 School of Chinese Materia Medica, Guangdong Pharmaceutical University, Guangzhou, China; 2 Key Laboratory of Lingnan Medicinal Materials Production and Development, National Administration of Traditional Chinese Medicine, Guangzhou, China; 3 Guangzhou Comprehensive Experiment Station of National Chinese Medicinal Materials Industry Technology System, Yunfu, China; 4 Institute of Forestry and Landscape Architecture, Guangzhou, China; 5 Teaching Center of Traditional Chinese Medicine, Guangdong Pharmaceutical University, Yunfu, China

**Keywords:** genetic diversity, germplasm resources, population structure, *Rhododendron mariae* Hance, SSR molecular markers, Phenotypic traits

## Abstract

**Introduction:**

Rhododendron mariae Hance is a traditional medicinal plant in the Lingnan region widely used for treating respiratory diseases. However, its wild germplasm resources are threatened by overharvesting and habitat destruction. To provide a scientific basis for conservation strategies, this study systematically investigated the genetic diversity and population structure of wild R. mariae populations.

**Methods:**

We analyzed eight wild populations in Guangdong Province by integrating phenotypic traits and SSR molecular markers. Phenotypic diversity was assessed using the coefficient of variation (CV), nested analysis of variance, and Neighbor-Joining (NJ) clustering. Concurrently, SSR markers were employed to evaluate genetic diversity parameters (including number of alleles and Shannon’s information index), and population structure was further elucidated through genetic distance clustering, Principal Component Analysis (PCA), and Structure analysis.

**Results:**

Phenotypic variation was primarily attributed to differences among populations (intraclass correlation coefficient ICC > 0.7 for all traits except leaf area). The GLS (CV = 0.26) and FLS (CV = 0.20) populations exhibited the most prominent phenotypic diversity. Molecular analysis confirmed high population differentiation (Fst = 0.1963). The DJS, FLS, XXC, and GLS populations displayed significantly higher genetic diversity parameters, identifying them as core germplasm resources. Both Structure and PCA analyses supported the division into three genetic subgroups, with GLS and XXC being highly differentiated populations, while others showed genetic admixture, highlighting the role of geographical isolation

**Conclusion:**

Integrating both lines of evidence, the FLS, GLS, XXC, and DJS populations were identified as priority conservation units due to their rich genetic diversity and uniqueness at both phenotypic and molecular levels. This study provides a critical scientific foundation for the conservation and sustainable utilization of wild R. mariae germplasm resources.

## 1 Introduction

*Rhododendron mariae* Hance, commonly known as *Mary’s Rhododendron*, is a perennial shrub belonging to the genus *Rhododendron* L. in the family Ericaceae. It is primarily distributed in tropical and subtropical regions. The genus exhibits extremely high global diversity, encompassing over a thousand species and numerous horticultural varieties. Based on ecotype, the genus *Rhododendron* can be divided into five subgenera: *Hymenanthes* (evergreen rhododendrons), *Rhododendron* (scaly rhododendrons), *Azaleastrum*, *Pentanthera*, and *Therorhodion* (Fang, 1999). It is noteworthy that *R. mariae* is the only species included in the 1977 edition of the Pharmacopoeia of the People’s Republic of China, valued for both its ornamental appeal and significant medicinal properties (Xia et al., 2021).Its young branches and leaves are rich in flavonoids, making it a traditional remedy for respiratory diseases such as asthma and bronchitis (Zeng, 2019; Wang, 2020; Zeng, 2021).It has been developed into commercial products including tablets, capsules, and expectorant cough granules (Chen et al., 2022).

Despite its significant value, current research has primarily focused on phytochemical analysis (Guo et al., 2014), leaving a considerable knowledge gap regarding the genetic background of its wild germplasm resources, population structure, and evidence-based conservation strategies. Furthermore, heavy reliance on wild harvesting coupled with habitat destruction poses a serious threat to the sustainable utilization of this species. *R. mariae* demonstrates broad ecological adaptability, abundant germplasm resources, and remarkable diversity in floral and leaf traits. Although these morphological characteristics are influenced by both genetic and environmental factors, they provide a comprehensive and intuitive reflection of germplasm properties, underscoring the importance of morphological traits in the classification of *R. mariae*. Researchers have employed diversity analysis, correlation analysis, principal component analysis (PCA), and cluster analysis to quantitatively evaluate phenotypic traits. For instance, Wang et al. (2025) conducted analysis of variance (ANOVA), coefficient of variation (CV) analysis, and cluster analysis on germplasm of *R. dauricum*, successfully categorizing materials into distinct variety groups and identifying germplasm with high ornamental and horticultural value. These evaluations revealed significant differentiation in morphological traits, with quantitative traits exhibiting greater diversity than qualitative traits.

Molecular markers, being independent of morphological traits and environmental conditions, can reveal genetic variation at the DNA level, thereby enabling a more precise analysis of the genetic diversity and interspecific relationships within *R. mariae*. Commonly used markers in genetic diversity studies include ISSR (Mahgoub et al., 2025), RAPD (Tang et al., 2025), and SSR (Zhang et al., 2025; Delmas et al., 2011; Pan et al., 2019).For instance, Delmas et al. (2011) and Pan et al. (2019) utilized next-generation sequencing technologies to isolate microsatellite loci in *R. ferrugineum L.* and *R. shanii Fang*, respectively, and designed primers for amplification. The average number of alleles per locus ranged from 1 to 20. Wu (2015) employed 14 SSR markers to analyze the genetic structure, diversity, and historical dynamics of two remnant populations of the extremely small population wild plant *R. protistum* var. giganteum, proposing conservation recommendations. Simple sequence repeats (SSRs) are considered one of the most important molecular markers. As heritable, co-dominant markers, SSRs can accurately discriminate differences between germplasms, enable rapid variety identification, and effectively overcome the limitations of traditional phenotypic analysis influenced by the environment. They are widely applied in plant genetic breeding and phylogenetic studies (Bayazıt et al., 2025; Luo et al., 2025).

In Guangdong Province, the type locality and one of the main distribution areas of *R. mariae*, the pattern of genetic variation among different geographical populations remains unclear, leading to a lack of targeted conservation strategies. This study integrates phenotypic traits and SSR molecular markers to systematically analyze the patterns of population differentiation in *R. mariae* from both morphological and genetic perspectives. It aims to identify genetic diversity hotspots and priority conservation units, thereby providing a scientific basis for formulating effective germplasm resource conservation strategies and promoting standardized cultivation.

## 2 Materials and methods

### 2.1 Wild population survey and sampling strategy

**Figure 1:**
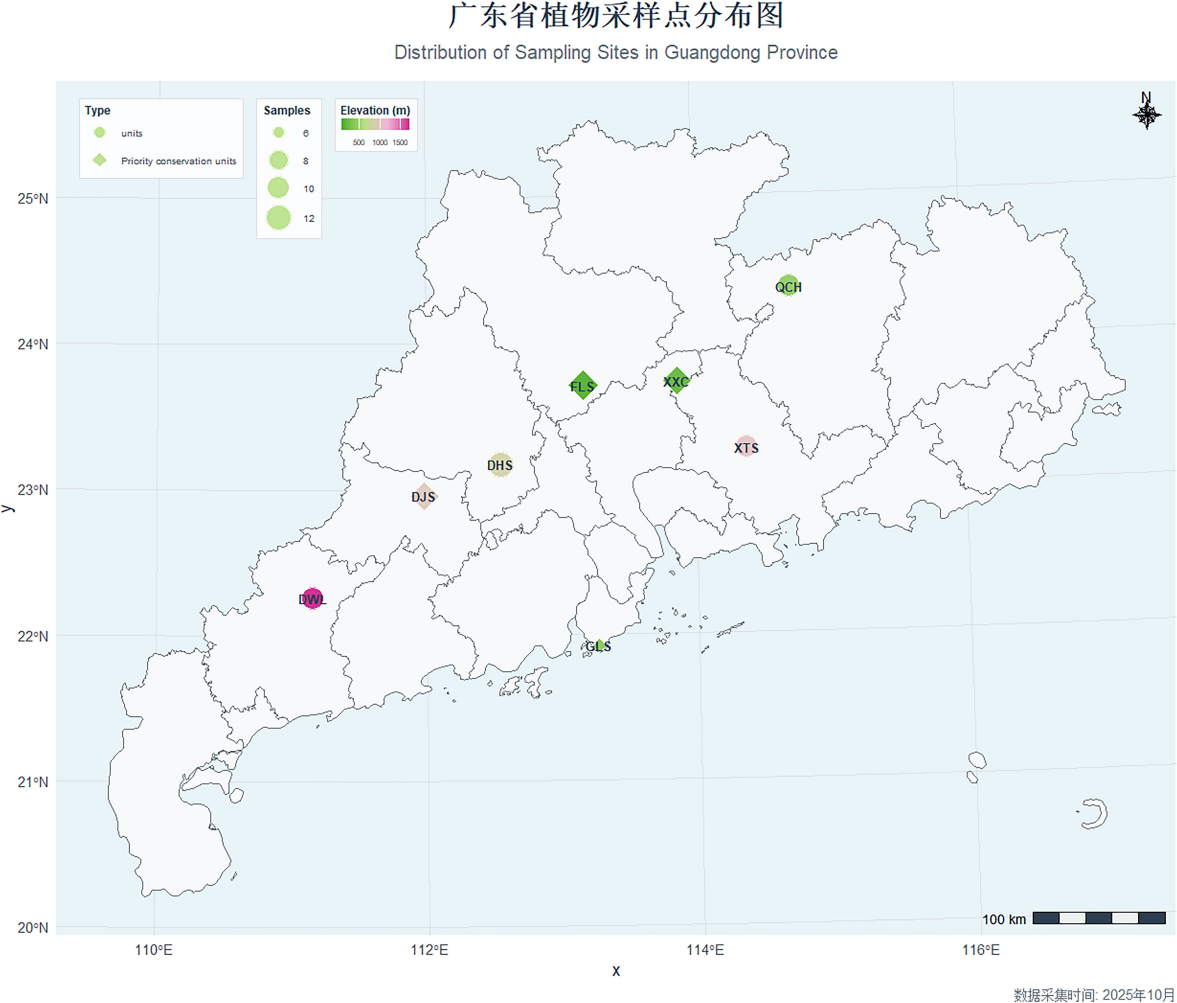
Sampling map of *Rhododendron mariae* populations in Guangdong Province.

Based on a comprehensive review of literature, specimen records from the Herbarium of South China Botanical Garden, Chinese Academy of Sciences, the Chinese Virtual Herbarium (CVH, 2024), and GPS coordinates obtained from preliminary resource surveys conducted by our research group (Jiang, 2022), eight representative wild populations of *R. mariae* across Guangdong Province were selected for this study (**Figure 1**). The sampling sites encompassed diverse altitudinal gradients from coastal lowlands to mountainous regions, thereby comprehensively reflecting the geomorphological diversity of Guangdong Province (**Table 1**). Within each population, sampling was conducted following a criterion of maintaining a minimum interplant distance of ≥50 m to minimize the collection of closely related individuals and avoid redundant sampling. Ultimately, a total of 78 *R. mariae* germplasm accessions were collected.

**Table 1:** Source information of different *Rhododendron mariae populations.*.

Location	Samples collected	Longitude (lng)	Latitude (lat)	Altitude (m)	Landform	Abbreviation
Dinghushan, Dinghu Dist., Zhaoqing	12	112.54533	23.17003	833	Montane Evergreen Broad-Leaved Forest	DHS
Dajinshan, Yun’an Dist., Yunfu	10	111.98199	22.95754	961.7	Montane Thicket	DJS
Xiaxiacun, Conghua Dist., Guangzhou	10	113.86982	23.72584	158	Valley Thicket	XXC
Qincaihu, Lianping County, Heyuan	10	114.71642	24.356989	399.2	Montane Thicket	QCH
Feilaisi, Qingcheng Dist., Qingyuan	10	113.16712	23.70266	80.1	Valley Thicket	FLS
Xiangtoushan, Boluo County, Huizhou	11	114.37651	23.26217	1024	Montane Evergreen Broad-Leaved Forest	XTS
Xiaosanlang, Gaolan Mountain, Zhuhai	6	113.26360	21.92086	333.6	Coastal Thicket	GLS
Dawuling, Xinyi City, Maoming	10	111.15700	22.25861	1703.8	Alpine Thicket	DWL

**Figure 2:**
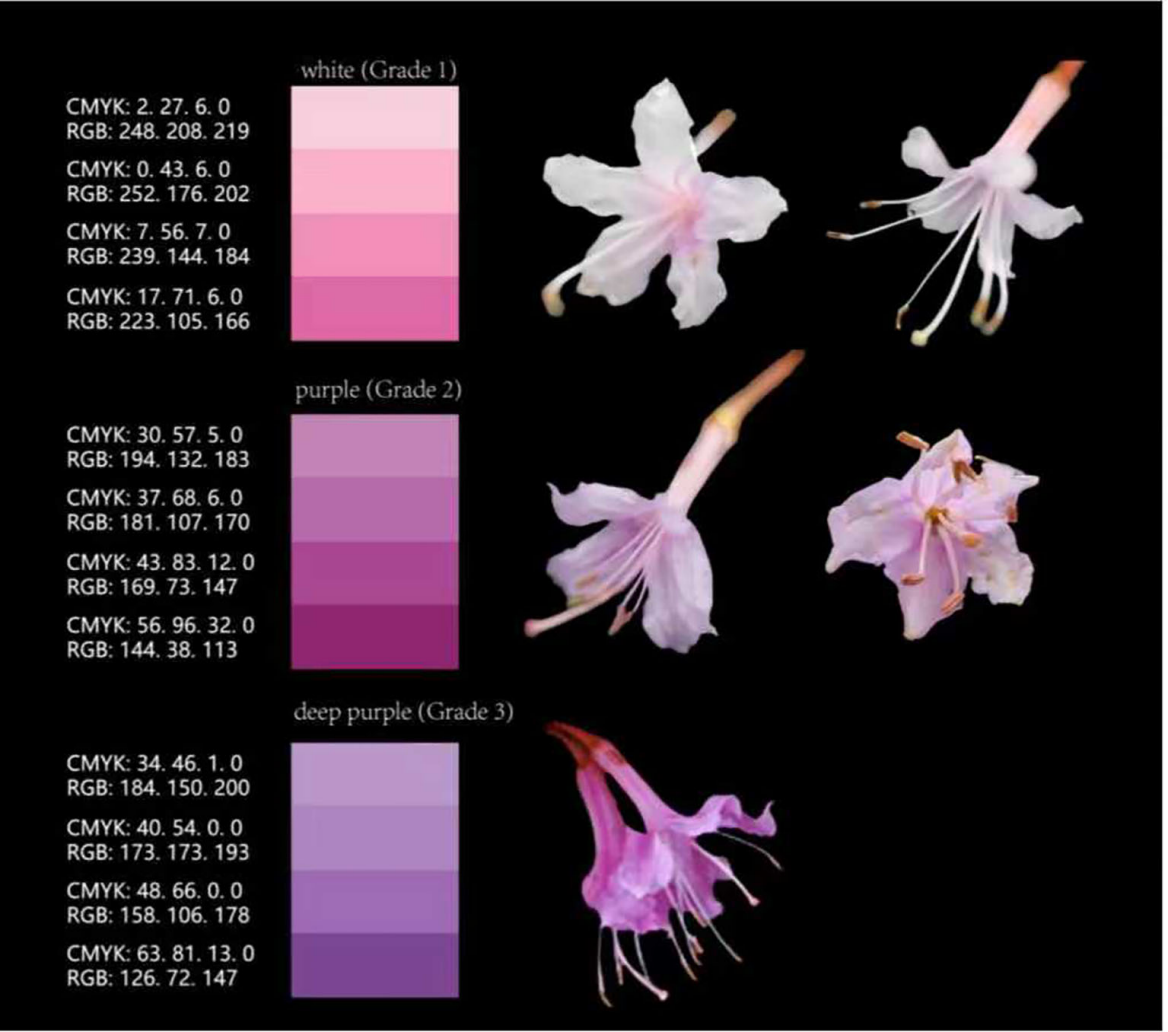
Grading of flower color phenotypic variation in *Rhododendron mariae.*.

Significant variation in phenotypic traits of *R. mariae* was observed during field investigations. Consequently, a digital caliper or leaf area meter was employed to quantify leaf and floral characteristics. The sampling protocol was as follows: within each population, six individual plants were selected, and the second fully expanded leaf from each plant was measured for leaf related traits. Simultaneously, three normally developed flowers were collected from plants across the populations for the measurement of floral traits. Based on the observed flower color variation and referring to the specific color scale parameters attached on the left side, the tested plants were categorized into three grades (**Figure 2**): white (Grade 1), purple (Grade 2), and deep purple (Grade 3).and deep purple (Grade 3) (Wang et al., 2025). A total of 11 phenotypic traits were measured, including flower color, stamen length, pistil length, flower diameter, leaf area, petiole length, pedicel length, leaf length, leaf width, and the leaf length-to-width ratio. The color standards for grading were defined using CMYK and RGB values as follows: red-purple (Grade 1) corresponds to CMYK (2.27%, 6.0%, 0%, 0%) – (17.7%, 16.6%, 0%, 0%) and RGB (248, 208, 219) – (223, 105, 166); purple (Grade 2) corresponds to CMYK (30.57%, 5.0%, 0%, 0%) – (56.96%, 32.0%, 0%, 0%) and RGB (194, 132, 183) – (144, 38, 113); deep purple (Grade 3) corresponds to CMYK (34.46%, 1.0%, 0%, 0%) – (63.81%, 13.0%, 0%, 0%) and RGB (184, 150, 200) – (126, 72, 147). CMYK represents the percentage composition of Cyan, Magenta, Yellow, and Key (black) ink used in color printing, while RGB denotes the intensity values (ranging from 0 to 255) of Red, Green, and Blue light channels in digital imaging. This dual-model color scale was employed to standardize visual assessment of floral color phenotypes under field conditions.

### 2.2 SSR marker-based analysis of wild population structure

#### 2.2.1 SSR primer screening, PCR amplification, and genotyping

Genomic DNA was extracted using a modified CTAB method (Yuan et al., 2007; Wan M. et al., 2025; Orazov et al., 2024). The specific procedure was as follows: fresh leaves (3–4 cm in length) were collected in the field and immediately placed into silica gel-filled tea bags for dehydration and preservation. Upon returning to the laboratory, samples were transferred to a -80 °C freezer for long-term storage. A total of 78 samples from the 8 populations were obtained for DNA extraction.

Sixty-eight SSR primer pairs, previously developed for closely related species (Luo et al., 2017; Yao et al., 2025), were initially screened. During the preliminary screening phase, two randomly selected individuals from each of the 8 populations were used for amplification tests. Ultimately, primer pairs that exhibited stable polymorphism, clear and reproducible bands were selected (primer details provided in **Supplementary Table 3**) for genotyping all samples.

The total volume of the PCR amplification reaction was 20 μL. The amplification program was set as follows: initial denaturation at 94 °C for 5 min; followed by 35 cycles of denaturation at 94 °C for 30 s, annealing at 56 °C for 30 s, and extension at 72 °C for 1 min; a final extension at 72 °C for 5 min; and hold at 4 °C. The amplification products were separated via 8%(w/v) polyacrylamide gel electrophoresis (120 V, 120 min). Bands were scored manually: the presence of a clear band at the target position was scored as ‘1’, while the absence of a band or a faint/unclear band was scored as ‘0’. This process resulted in the construction of a binary SSR band matrix (Orazov et al., 2024; Zhang et al., 2016; Beghè et al., 2024).

### 2.3 Statistical analysis

#### 2.3.1 Phenotypic data analysis

Phenotypic data were initially organized using Microsoft Excel 2019. General Linear Model (GLM) multivariate analysis was performed using IBM SPSS Statistics 19.0 (Liu et al., 2025; Zhang et al., 2007). The coefficient of variation (CV) was calculated to quantify the dispersion of traits using the formula: CV = (Standard Deviation/Mean) × 100% (Chen et al., 2016; Petruccelli et al., 2025). Considering the significant effect of within-population variation on the results, a mixed linear model (MLM) with a two-factor nested design was employed for analysis of variance (ANOVA) instead of the classical GLM, implemented using R software version 4.3.3 (M. Wan et al., 2025). Cluster analysis based on phenotypic data was performed using the Unweighted Pair Group Method with Arithmetic Mean (UPGMA) (Wan T. et al., 2025), and a phylogenetic tree was constructed using the Neighbor-Joining (NJ) method based on Euclidean distances (Copley et al., 2024; Chen et al., 2016).A neighbor-joining (NJ) phylogenetic tree was constructed based on Nei’s genetic distance using R software version 4.3.3,with 1000 bootstrap replicates to assess branch support.

#### 2.3.2 SSR marker data analysis

The SSR binary matrix was analyzed using Popgene 32, PowerMarker, and GenAlEx software to calculate genetic diversity parameters (Petruccelli et al., 2025; Xie et al., 2006). These parameters included the number of alleles (Na), effective number of alleles (Ne), observed heterozygosity (Ho), expected heterozygosity (He), Shannon’s Information Index (I), percentage of polymorphic loci (PPL), Nei’s gene diversity index (H), gene flow (Nm), and the fixation index (Fst) (Kumar et al., 2019). The chi-square test was used to assess statistical significance.

Population genetic structure was analyzed using Structure software version 2.3.4 (Singh et al., 2020; Sharma et al., 2024; Ibrar et al., 2022).First, the SSR band data was converted into a binary matrix (presence=1, absence=0, missing data=9) and subsequently transformed into the Structure input format using DataFormater software. Analysis parameters were set as follows: burn-in period of 5,000 iterations, Markov Chain Monte Carlo (MCMC) chain length of 10,000 repetitions after burn-in, a presumed number of genetic clusters (K) ranging from 1 to 10, with 10 independent runs for each K value. The optimal number of clusters (K) was determined by calculating the ΔK statistic using the online tool Structure Selector; K = 3 was identified as the optimal value corresponding to the highest ΔK). The final results were visualized using CLUMPAK software.

## 3 Results

### 3.1 Phenotypic diversity analysis

#### 3.1.1 Coefficient of variation of phenotypic traits

To elucidate the phenotypic diversity among different populations, this study measured 11 phenotypic traits across 48 samples and calculated the coefficient of variation (CV, **Table 2**). Analysis at the population level revealed that the GLS (CV = 0.26) and FLS (CV = 0.20) populations exhibited the highest degree of phenotypic variation, classifying them as high-variability groups. This suggests they harbor rich phenotypic diversity and possess high value for resource selection. In contrast, low-variability groups such as DJS and XXC showed higher phenotypic uniformity, potentially indicating relatively stable habitats or exposure to stronger environmental selection pressures.

**Table 2:** Coefficient of variation (CV) for 11 phenotypic traits in different populations of *Rhododendron mariae* in Guangdong Province.

Population	Leaf length	Leaf width	Leaf area	Leaf length/width ratio	Petiole length	Stamen length	Pistil length	Corolla length	Corolla lobe length	Pedicel length	Flower color	Meancv
XXC	0.07	0.13	0.09	0.15	0.07	0.03	0.15	0.11	0.15	0.29	0	0.11
DHS	0.14	0.07	0.2	0.09	0.14	0.04	0.05	0.08	0.13	0.13	0.34	0.13
QCH	0.07	0.13	0.18	0.1	0.09	0.07	0.07	0.08	0.16	0.06	0.3	0.12
FLS	0.19	0.24	0.39	0.22	0.25	0.07	0.15	0.05	0.18	0.09	0.35	**0.20**
XTS	0.21	0.19	0.36	0.07	0.11	0.09	0.18	0.03	0.06	0.1	0.17	0.14
GLS	0.41	0.29	0.64	0.16	0.26	0.12	0.11	0.12	0.3	0.17	0.25	**0.26**
DWL	0.17	0.19	0.37	0.06	0.21	0.11	0.16	0.06	0.16	0.07	0.31	**0.17**
DJS	0.1	0.13	0.17	0.15	0.13	0.03	0.05	0.14	0.19	0.16	0	0.11
**Meancv**	**0.17**	**0.17**	**0.30**	**0.13**	**0.16**	**0.07**	**0.12**	**0.08**	**0.17**	**0.13**	**0.22**	**0.16**

*Note: CV = (Standard Deviation/Mean) × 100%, reflects the degree of trait variation; meancv: Average of cv*.

Bold font represents the average values.

At the trait level, the 11 phenotypic traits were ranked by CV from high to low as follows: leaf area > leaf width > leaf length > flower color > pedicel length > corolla lobe length (**Table 2**). Leaf area displayed the highest CV (0.30), while flower color had a CV of 0.22, a difference of 0.08, indicating significant variation in the degree of differentiation among traits within populations. Notably, inter-population variation in flower color showed considerable fluctuation: the QCH population, where individuals with two or more flower colors were collected, had a high CV for this trait, whereas the DJS population, where only one flower color was observed, had a significantly lower CV.

Nested analysis of variance (**Table 3**) showed that for 8 of the 11 phenotypic traits, including leaf length-to-width ratio, petiole length, and stamen length, the ICC was > 0.7. The ICC for leaf length, leaf width, and leaf area was also > 0.5. This indicates that variation in the vast majority of traits is predominantly driven by differences between populations, revealing significant differentiation among them. F-statistic results were ranked as follows: corolla length (22.51) > stamen length (16.94) > corolla lobe length (16.51) > leaf length-to-width ratio (13.70) > pistil length (13.58) > leaf width (13.18) > leaf length (11.88) > pedicel length (11.30) > petiole length (10.98). The P value for all traits were < 0.0001, confirming the extremely high statistical significance of phenotypic differences among populations.

**Table 3:** Nested analysis of variance for 11 phenotypic traits of *Rhododendron mariae*.

Traits	ICC	F	DF	P	95% confidence interval
Leaf length	0.6	11.88	8.08	2.11E-06	4.12-6.10
Leaf width	0.57	13.18	7.96	1.09E-06	1.80-2.57
Leaf area	0.52	7.27	8.01	8.60E-05	5.68-10.96
Leaf length/width ratio	0.72	13.7	8.02	7.61E-07	1.97-2.77
Petiole length	0.74	10.98	7.76	5.34E-06	0.57-0.88
Stamen length	0.85	16.94	7.97	1.57E-07	2.18-2.87
Pistil length	0.87	13.58	7.92	9.12E-07	2.40-3.38
Corolla length	0.83	22.51	7.95	1.75E-08	2.12-2.61
Corolla lobe length	0.77	16.51	7.95	1.96E-07	1.03-1.36
Pedicel length	0.84	11.3	8	3.40E-06	0.79-1.21
Flower color	0.55	8.59	7.97	2.67E-05	1.24-2.15

*Note: ICC denotes the intraclass correlation coefficient, reflecting the proportion of total variation attributable to differences between groups (ICC > 0.7 indicates inter-group variation dominates, < 0.7 indicates higher contribution of intra-group variation); P < 0.001 indicates highly significant differences, demonstrating the statistical significance of trait variation distribution among populations.*.

Further analysis revealed a clear distinction in the sources of trait variation: floral traits (e.g., stamen length, corolla length) exhibited a greater contribution from inter-population variation (ICC generally > 0.8) and were significantly influenced by geographical regional differences. Among leaf traits, leaf area showed the weakest inter-population effect (ICC = 0.52), with relatively prominent differences among individuals within groups, suggesting lower susceptibility to environmental modification and higher genetic stability. This result indicates that leaf morphological characteristics can serve as core indicators reflecting the genetic diversity of *R. mariae*, while phenotypic differentiation of floral traits is more readily influenced by environmental factors.

#### 3.1.2 Phylogenetic analysis based on phenotypic traits

**Figure 3:**
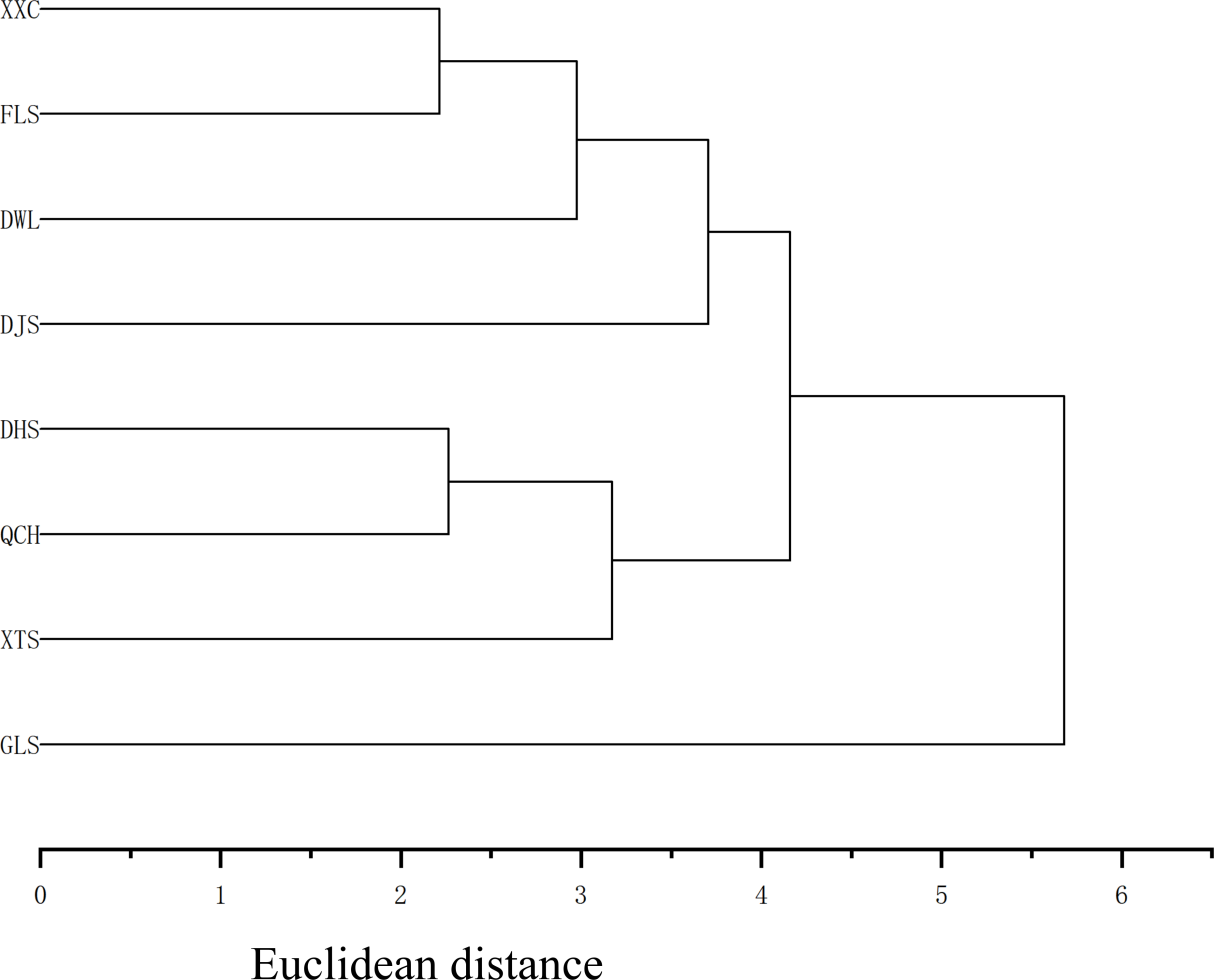
Cluster analysis of Rhododen*dron mariae* populations in Guangdong Province based on phenotypic traits.

After normalizing the continuous phenotypic data of 11 traits from *Rhododendron mariae Hance* in different regions of Guangdong Province, cluster analysis was performed based on Euclidean distance, and the results are shown in **Figure 3**.

Based on Neighbor-Joining (NJ) cluster analysis of phenotypic traits, the eight *R. mariae* populations were classified into three major groups (**Figure 3**). Group I comprised the XXC, FLS, DJS, GLS, and DWL populations. The high similarity in genetic background among these populations suggests a potential shared ancestral origin or historical gene flow during evolution, leading to convergent phenotypic characteristics. Group II consisted solely of the XTS population. Its independent clustering implies that this population may have undergone unique local adaptive evolution or long-term geographical isolation, resulting in a distinct genetic structure. Group III included the DHS and QCH populations, both of which exhibited relatively low genetic diversity (**Table 4**), potentially attributable to habitat homogenization or genetic drift leading to weak phenotypic differentiation. Notably, the DHS and QCH populations clustered together with the highest bootstrap support value of 85.4%, indicating highly similar gene pools. Frequent gene flow is hypothesized to be a major driver of their high phenotypic consistency, corroborating the low inter-population variation (intraclass correlation coefficient > 0.8, **Table 3**) observed for floral traits (e.g., stamen length, corolla length) in the nested ANOVA.

**Table 4:** Genetic diversity parameters of 8 *Rhododendron mariae* populations in Guangdong Province based on SSR markers.

Population	Number of samples	MEAN	PPL	NA	NE	I	(Obs_Het)	(Nei’sHet)
GLS	6	8	90.00%	**2.40 ± 0.97**	**1.88 ± 0.45**	**0.69 ± 0.31**	**0.50 ± 0.31**	**0.43 ± 0.17**
XXC	10	16	100.00%	**2.10 ± 0.32**	**1.94 ± 0.21**	**0.69 ± 0.10**	**0.73 ± 0.24**	**0.48 ± 0.06**
DJS	10	17	100.00%	**3.30 ± 0.95**	**2.60 ± 0.70**	**1.00 ± 0.29**	**0.79 ± 0.20**	**0.59 ± 0.12**
QCH	10	17	90.00%	2.22 ± 0.44	1.85 ± 0.35	0.66 ± 0.17	0.69 ± 0.27	0.44 ± 0.12
DHS	12	17	100.00%	2.00 ± 0.00	1.78 ± 0.28	0.61 ± 0.13	0.60 ± 0.34	0.42 ± 0.11
XTS	11	18	90.00%	2.00 ± 0.00	1.84 ± 0.16	0.64 ± 0.06	0.66 ± 0.16	0.45 ± 0.05
DWL	10	17	90.00%	2.00 ± 0.00	1.76 ± 0.31	0.60 ± 0.13	0.69 ± 0.32	0.41 ± 0.12
FLS	10	16	90.00%	**2.33 ± 0.71**	**2.00 ± 0.73**	**0.69 ± 0.32**	**0.64 ± 0.32**	**0.44 ± 0.18**

*(Note: Ensure **Table 4** content is translated. Include definitions: Na, Number of different alleles; Ne, Effective number of alleles=1/(Sum pi^2); I, Shannon’s Information Index=-1*Sum(pi*Ln(pi)); Ho, Observed heterozygosity=Number of Heterozygotes/N; He, Expected heterozygosity=1-Sum pi^2; uHe, Unbiased expected heterozygosity=(2N/(2N-1))*He)*.

Bold font in the table indicates the four populations with higher genetic diversity.

### 3.2 Genetic diversity and structure analysis based on SSR markers

#### 3.2.1 Genetic diversity parameters

Genetic diversity analysis of the 8 *R. mariae* populations (78 samples) (**Table 4**) revealed a high mean number of allele loci, indicating that the species overall retains rich allelic resources and genetic diversity. At the population level, the percentage of polymorphic loci (PPL) ranged from 90.00% to 100%. The genetic diversity parameters (number of alleles, Na; effective number of alleles, Ne; Shannon’s Information Index, I; expected heterozygosity, He) for the DJS, FLS, GLS, and XXC populations were significantly higher than those of other groups, identifying them as core reservoirs of genetic diversity with priority for conservation.

Notably, the observed heterozygosity (Ho) exceeded the expected heterozygosity (He) in all populations (e.g. XXC Ho=0.73 > He=0.48), characterizing a heterozygote excess and a deviation from Hardy-Weinberg equilibrium. This phenomenon may stem from recent population admixture or natural selection favoring heterozygotes, reflecting the development of a heterozygote advantage strategy in the species adaptive evolution. Moreover, this phenomenon is not uncommon Luo et al. (2025) also reported similar issues in their study of Ericaceae plants in Sichuan Province, which may be attributed to the limitations of intra-provincial surveys or intrinsic characteristics of *Rhododendron* species. Analysis of F-statistics (**Table 5**) showed that the mean inbreeding coefficient (Fis) across the 10 SSR loci was -0.4321 (range: -0.7070 to -0.1587). All loci exhibited significant heterozygote excess. Combined with experimental reproducibility validation (stable primer polymorphism, consistent band scoring), technical artifacts were ruled out, confirming that directional natural selection favoring heterozygotes is the primary cause. This reflects the evolution of a heterozygote advantage strategy in the species adaptation.

**Table 5:** F-statistics and gene flow analysis of *Rhododendron mariae* populations in Guangdong Province based on 10 SSR. primers.

Locus	Sample size	Fis	Fit	Fst	Nm*
78	134	-0.3403	-0.1546	0.1386	1.5537
910	132	-0.5672	-0.4290	0.0882	2.5855
1112	142	-0.5619	-0.4450	0.0748	3.0902
1314	144	-0.4791	-0.3937	0.0577	4.0798
1718	146	-0.3303	-0.0438	0.2153	0.911
1920	114	-0.1587	0.2099	0.3182	0.5358
2122	142	-0.2906	-0.1140	0.1369	1.5761
2324	154	-0.4435	-0.2083	0.1629	1.2847
2526	62	-0.707	0.4584	0.6827	0.1162
12	150	-0.5429	-0.3729	0.1102	2.0183
Mean	132	**-0.4321**	**-0.1510**	**0.1963**	**1.0235**

*(Note: Ensure **Table 5** content is translated. Include definition: Nm, gene flow estimate ≈ 0.25(1 - Fst)/Fst)*.

Bold font represents the average values.

The average inter-population genetic differentiation coefficient (Fst) was 0.1963, indicating that 19.63% of the total genetic variation originates from differentiation between populations. According to standard classification (Fst > 0.15 indicates high differentiation), this value is at a medium-high level, confirming that the 8 geographical populations have developed significant genetic structure differentiation. This aligns with the high inter-population variation observed in phenotypic traits (ICC > 0.7, **Table 3**).

The average gene flow (Nm) was 1.0235, indicating limited but effective gene exchange between populations. While its intensity is insufficient to completely counteract differentiation caused by genetic drift and natural selection, it maintains basic genetic connectivity among populations, preventing the complete formation of reproductive isolation.

Thus, the population genetic structure of *R. mariae* exhibits three main characteristics: (1) Medium-high genetic differentiation: Fst=0.1963 indicates the species has differentiated into subpopulations with significant genetic structure differences, and genetic variation is primarily contributed from within populations; (2) Heterozygote selection advantage: Fis=-0.4321 reflects a widespread heterozygote excess within populations, suggesting the preferential retention of heterozygotes by natural selection, which is an important genetic mechanism for maintaining population adaptive potential; (3) Restricted gene flow: A gene flow level of Nm≈1 promotes population continuity while allowing the accumulation of local adaptive differentiation, ultimately forming a genetic pattern of “overall continuity with local differentiation”.

#### 3.2.2 Genetic relationship and principal component analysis

**Figure 4:**
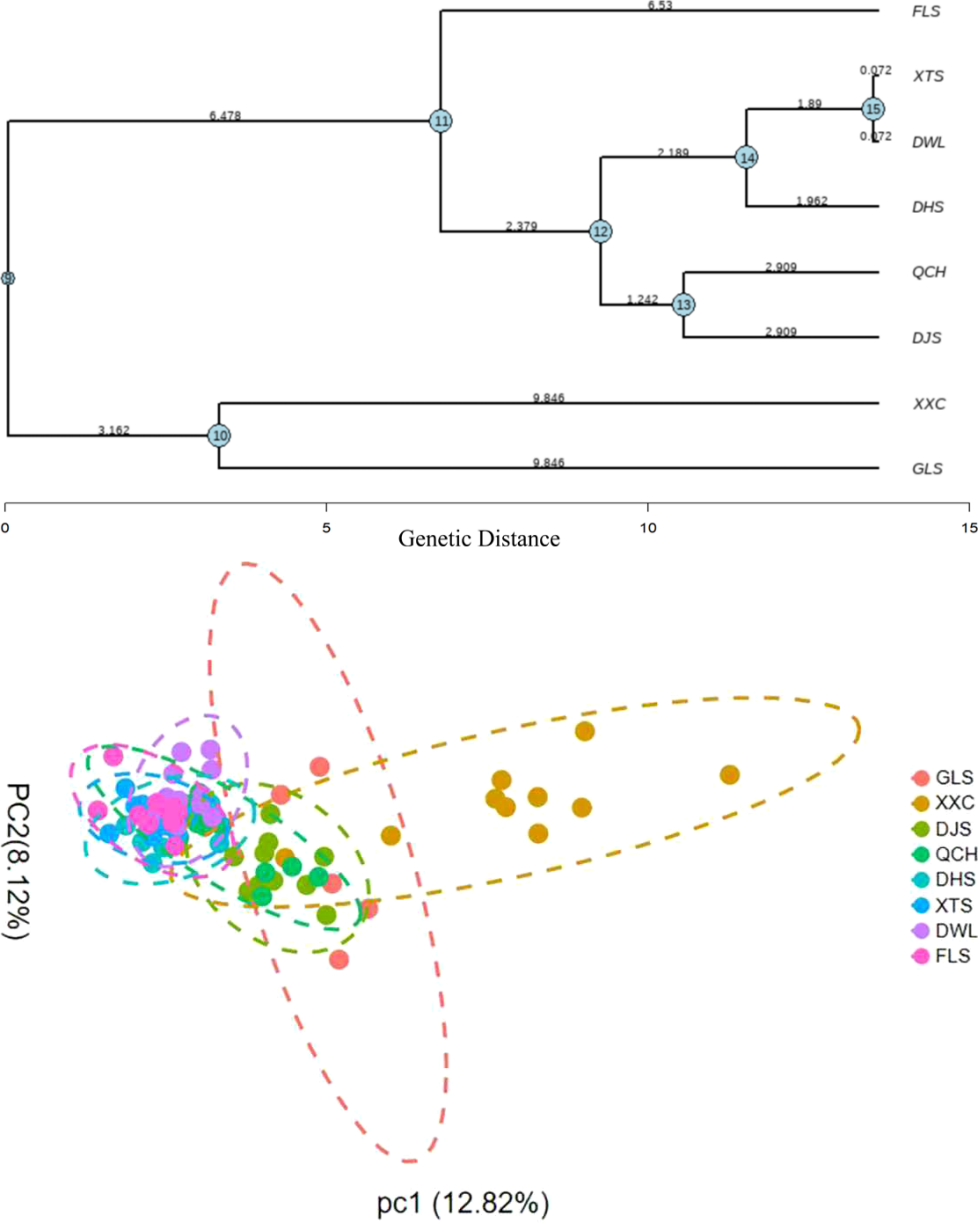
UPGMA dendrogram based on Nei’s genetic distance and Principal Component Analysis (PCA) of *Rhododendron mariae* populations based on SSR markers. Bold text represents genetic similarity, and blue text represents bootstrap values.

Genetic analysis using SSR markers further revealed the diversity and structural characteristics of the populations. A UPGMA dendrogram constructed based on Nei’s genetic distance matrix (genetic distance threshold of 2.379) divided the eight populations into four major clusters. This result was highly consistent with the clustering pattern from principal component analysis (PCA), collectively confirming significant genetic structure differentiation among the populations (**Figure 4**). Specifically, Cluster I consisted solely of the FLS population, which had a genetic branch length of (6.53) and showed highly differentiated characteristics, suggesting that this population may have experienced strong genetic drift or long-term isolation. Cluster II encompassed the majority of samples and could be further subdivided into two subclusters: DWL and XTS, and DHS. Cluster III comprised the DJS and QCH populations, indicating close genetic relationships between them. Cluster IV included the XXC and GLS populations, demonstrating unique genetic backgrounds.

Inter-population genetic distances varied significantly. The shortest genetic distance (1.89) was observed between the DWL and XTS populations, identifying them as recently diverged sister populations with low genetic differentiation. In contrast, the GLS and XXC populations, clustered together due to their high genetic distinctiveness, are recommended for inclusion as priority conservation units.

PCA results further validated the aforementioned clustering pattern: the GLS and XXC populations showed minimal overlap in the PCA plot, indicating significant genetic differentiation. The distributions of the remaining populations exhibited substantial overlap, reflecting strong genetic similarity and close relatedness, which suggests extensive gene flow among these groups.

#### 3.2.3 Genetic structure analysis

**Figure 5:**
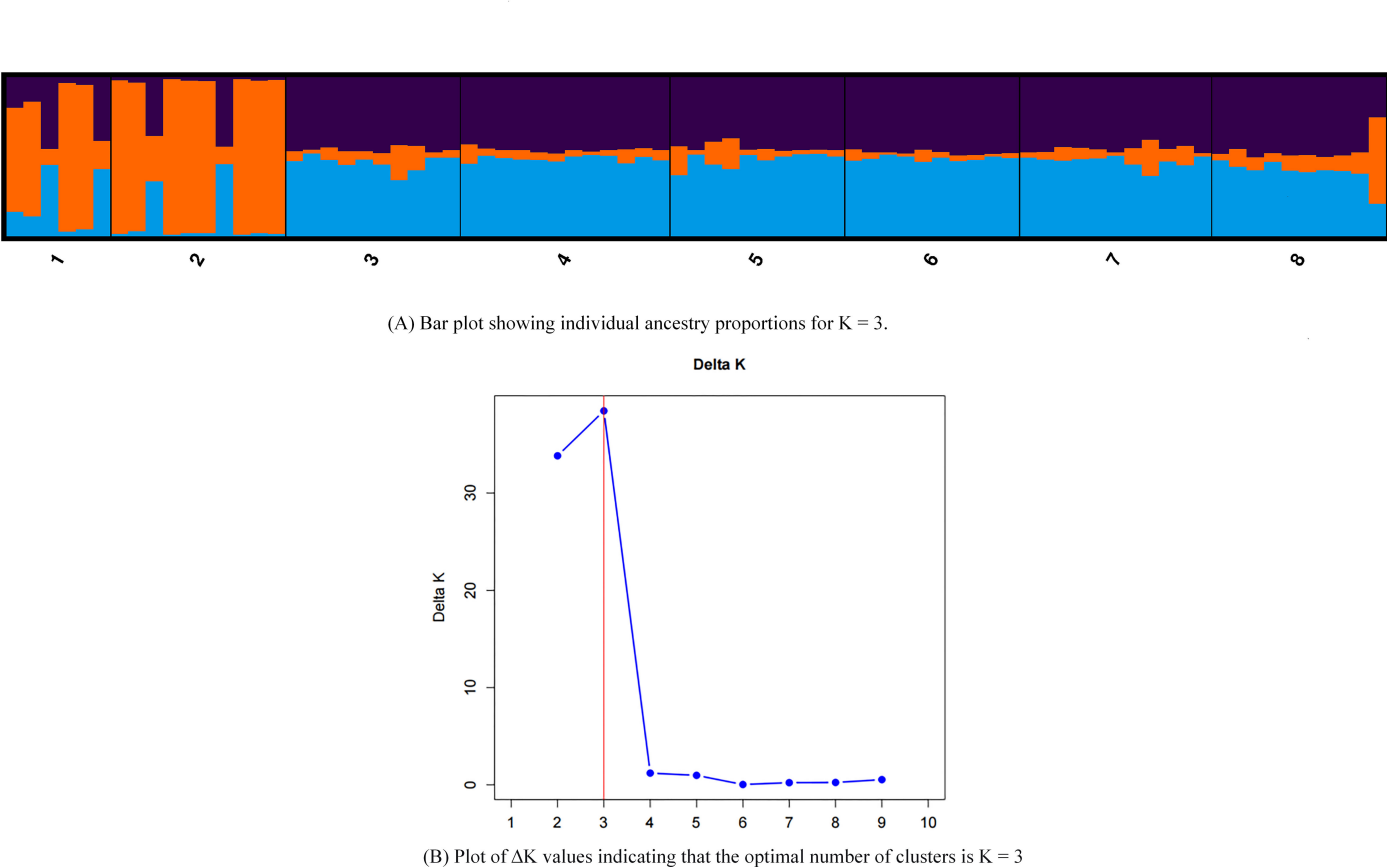
Genetic structure analysis of *Rhododendron mariae* populations in Guangdong Province based on SSR markers. **(A)** Bar plot showing individual ancestry proportions (Q matrices) for K = 3. **(B)** Plot of ΔK values indicating the optimal number of clusters is K = 3. (Populations 1-8: 1=GLS, 2=XXC, 3=DJS, 4=QCH, 5=DHS, 6=XTS, 7=DWL, 8=FLS).

The ΔK value calculated by StructureSelector peaked at K = 3 (**Figure 5**), supporting the division of all samples into three major genetic clusters. This result highly aligns with the divisions obtained from PCA and UPGMA cluster analysis (genetic distance threshold 3.162), confirming the presence of clear genetic structure differentiation within *R. mariae* populations in Guangdong Province. This differentiation pattern likely stems from the combined effects of geographical isolation (e.g., mountain barriers), local micro-environmental adaptive selection, and historical migration events.

Analysis based on the population genetic structure model (STRUCTURE) revealed that the genetic composition of all 78 samples exhibited admixture characteristics (multicolored stacking in the bar plot), indicating a history of extensive gene flow among different geographical populations within Guangdong Province. This gene flow, mediated either naturally or anthropogenically, is a crucial factor in maintaining the high level of genetic variation within populations, consistent with the conclusion from F-statistic analysis that “restricted gene flow (Nm≈1) can still maintain population continuity”.

Further analysis of the genetic composition revealed that *R. mariae* populations in Guangdong Province are divided into three groups: GLS, XXC, and other clusters. (This result is consistent with the previous PCA and cluster analysis.) Although GLS and XXC are classified into different groups, their genetic backgrounds share similarities: both exhibit a high proportion of heterozygotes. In contrast, other clusters show relatively fewer heterozygotes, which may be attributed to stronger anthropogenic influences in the Pearl River Delta region, leading to restricted gene exchange with other populations and consequently higher genetic uniqueness. The remaining clusters generally display complex admixed genetic backgrounds, reflecting frequent gene flow throughout their evolutionary history.

## 4 Discussion

### 4.1 Genetic diversity characteristics and conservation value

This study, through integrated analysis of phenotypic traits and SSR molecular markers, systematically reveals for the first time the genetic diversity characteristics of wild *R. mariae* populations in Guangdong Province. At the phenotypic level, the average coefficient of variation (CV) for the 11 traits was 0.16. The GLS (CV = 0.26) and FLS (CV = 0.20) (**Table 2**) populations exhibited significant phenotypic differentiation, reflecting their adaptive potential to heterogeneous habitats. This result is consistent with the nested ANOVA of leaf and floral traits, indicating that inter-population variation is the dominant factor in phenotypic differentiation, with floral traits (e.g., stamen length, corolla length, ICC > 0.8) being more significantly influenced by geographical isolation and environmental selection.

Molecular markers further verified the high genetic diversity of the species: the percentage of polymorphic loci (PPL) across the eight populations reached 90.00%–100%. Parameters such as the number of alleles (Na) and Shannon’s Information Index (I) were significantly higher in the DJS (He=0.59), FLS (He=0.44), GLS (He=0.43), and XXC (He=0.48) populations, identifying them as the core reservoirs of genetic diversity. Notably, all populations exhibited observed heterozygosity (Ho) higher than expected heterozygosity (He) (e.g., XXC Ho=0.73 > He=0.48). Combined with the Fis statistic of -0.4321, this confirms that directional natural selection favoring heterozygotes is an important mechanism for maintaining population adaptive potential. This heterozygote advantage synergizes with the high variability of phenotypic traits, providing a genetic foundation for *R. mariae* to respond to environmental fluctuations.

Integrating phenotypic and molecular evidence, the DJS, FLS, GLS, and XXC populations are identified as priority conservation units due to their rich phenotypic variation and genetic diversity. Their conservation value lies not only in maintaining the species’ evolutionary potential but also in providing high-quality germplasm for screening specific medicinal compounds (e.g., flavonoids), achieving a win-win scenario for resource conservation and sustainable utilization.

### 4.2 Drivers of population genetic differentiation

The genetic differentiation of wild *Rhododendron mariae* populations in Guangdong Province exhibits a unique pattern of “overall continuity with local differentiation.” Molecular markers revealed an inter-population genetic differentiation coefficient (Fst) of 0.1963 (>0.15), indicating that approximately 19.63% of the genetic variation stems from differentiation among geographical populations, reaching a medium-high level. Structure analysis supported the division of populations into three genetic subgroups (K = 3), which highly aligns with the UPGMA clustering (genetic distance threshold 3.162) and PCA results, confirming geographical isolation as a core driving factor for this differentiation.

Specifically, the genetic parameters (Nm = 1.02, Fst = 0.1963) revealed moderate population differentiation alongside measurable gene flow. The magnitude of gene flow (Nm > 1) is sufficient to counteract divergence, thereby sustaining genetic connectivity and contributing to an overall homogenization trend. This dynamic supports the observed pattern of “overall continuity with local differentiation” in Guangdong’s *Rhododendron mariae*. This differentiation pattern generally corresponds to geographical distribution—neighboring populations such as QCH and XTS, and DWL and XTS, clustered together preferentially. However, the trans-regional genetic similarity between DJS and DHS suggests that human activities (e.g., anthropogenic migration due to medicinal material harvesting) or animal pollination may breach geographical barriers, serving as auxiliary pathways for gene flow. Gene flow is primarily driven by natural mechanisms, such as insect pollination, wind, or water dispersal of seeds. Additionally, pollination by certain animals, as well as human activities, such as inadvertently carrying seeds or plant fragments during herb gathering, hiking, or transportation, can also facilitate genetic exchange across regions. These combined natural and anthropogenic pathways work together to enable genetic interchange even between geographically distant populations.

Discrepancies between phenotypic and molecular clustering results (e.g., the different grouping of XXC in the two types of analysis) further unveil the complexity of genetic differentiation: floral traits are susceptible to environmental modification, whereas SSR markers more stably reflect the genetic essence. This is consistent with the general pattern in the genus *Rhododendron*, where phenotypic plasticity and genetic differentiation jointly shape population structure.

### 4.3 Conservation strategy recommendations

The conservation of needs to balance the maintenance of genetic diversity and the enhancement of population connectivity. On one hand, *in-situ* conservation sites should be established for the DJS, FLS, GLS, and XXC populations, restricting human harvesting and habitat destruction. Particular attention should be paid to protecting the habitat of effective pollinators like bumblebees to mitigate pollination limitations caused by early spring rainy seasons (Tian et al., 2011; Zhang et al., 2004). On the other hand, based on phenotype-molecular association analysis, superior individuals with high medicinal compound content should be selected to establish a large-scale seedling propagation system. This will subsequently reduce pressure on wild populations through standardized cultivation.

This study clarifies the genetic diversity characteristics and identifies priority conservation units for wild *R. mariae* populations in Guangdong. Future research could integrate genomics to decipher adaptive evolution mechanisms and explore the association between medicinal compounds and genetic markers, laying the groundwork for elite cultivar breeding. Furthermore, a dynamic monitoring system should be established, coupled with ecological restoration technologies, to promote the recovery of wild populations and achieve a balance between resource conservation and sustainable utilization, providing a model for the conservation of traditional Lingnan medicinal plants.

## Data Availability

The datasets presented in this study can be found in online repositories. The names of the repository/repositories and accession number(s) can be found in the article/**Supplementary Material**.
