# Supplementary figures and images for: Integrated analysis of phenotypic and SSR data reveals the genetic structure differentiation of wild Rhododendron mariae Hance populations in Guangdong and the driving factors for conservation planning
Source: Front Plant Sci. 2026 Mar 23;17:1723036. doi: 10.3389/fpls.2026.1723036 (PMC13050912; doi:10.3389/fpls.2026.1723036)

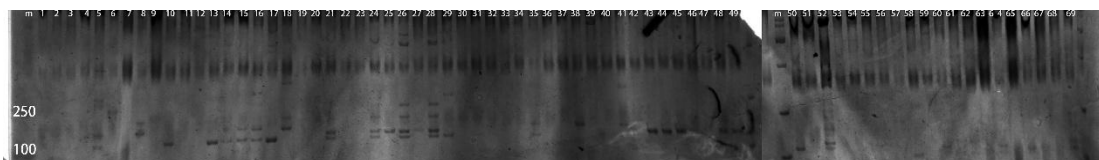

1-68:GLS XXC DHS QCH DWL XTS FLS

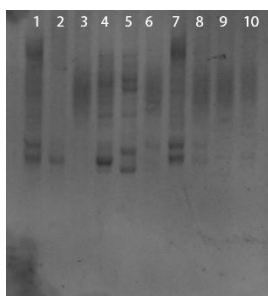

1-10 :DJS

Supplement: Supplementary file 1 [file DataSheet1.pdf]
