# Supplementary material for: Integrated analysis of phenotypic and SSR data reveals the genetic structure differentiation of wild Rhododendron mariae Hance populations in Guangdong and the driving factors for conservation planning
Source: Front Plant Sci. 2026 Mar 23;17:1723036. doi: 10.3389/fpls.2026.1723036 (PMC13050912; doi:10.3389/fpls.2026.1723036)
Supplement: Supplementary file 2 [file Table1.doc]

Nei's Unbiased Measures of Genetic Identity and Genetic distance [See Nei (1978) Genetics 89:583-590]

pop ID 1 2 3 4 5 6 7 8

===============================================================================

1 **** 0.8336 0.8213 0.7182 0.8168 0.7447 0.6821 0.7560

2 0.1820 **** 0.8794 0.9033 0.9435 0.9408 0.9163 0.8293

3 0.1969 0.1285 **** 0.7510 0.8424 0.8029 0.7494 0.7011

4 0.3310 0.1018 0.2863 **** 0.9303 0.9519 0.9712 0.8606

5 0.2023 0.0582 0.1716 0.0722 **** 0.9276 0.9043 0.8446

6 0.2948 0.0611 0.2195 0.0493 0.0751 **** 0.9986 0.9232

7 0.3826 0.0874 0.2885 0.0292 0.1006 0.0014 **** 0.9352

8 0.2797 0.1872 0.3551 0.1501 0.1689 0.0799 0.0670 ****

===============================================================================
